# Spatial Access and Willingness to Use Pre-Exposure Prophylaxis Among Black/African American Individuals in the United States: Cross-Sectional Survey

**DOI:** 10.2196/12405

**Published:** 2019-02-04

**Authors:** Bisola O Ojikutu, Laura M Bogart, Kenneth H Mayer, Thomas J Stopka, Patrick S Sullivan, Yusuf Ransome

**Affiliations:** 1 Department of Medicine, Brigham and Women's Hospital Division of Global Health Equity Harvard Medical School Boston, MA United States; 2 RAND Corporation Santa Monica, CA United States; 3 The Fenway Institute Boston, MA United States; 4 Department of Public Health and Community Medicine Tufts University School of Medicine Boston, MA United States; 5 Department of Epidemiology Emory Rollins School of Public Health Atlanta, GA United States; 6 Department of Social and Behavioral Sciences Yale School of Public Health New Haven, CT United States

**Keywords:** barriers, black individuals, HIV prevention, HIV services, pre-exposure prophylaxis, PrEP, racial disparities, spatial access

## Abstract

**Background:**

Uptake of pre-exposure prophylaxis (PrEP) among black individuals in the United States is low and may be associated with the limited availability of clinics where PrEP is prescribed.

**Objective:**

We aimed to determine the association between spatial access to clinics where PrEP is prescribed and willingness to use PrEP.

**Methods:**

We identified locations of clinics where PrEP is prescribed from AIDSVu.org and calculated the density of PrEP clinics per 10,000 residents according to the ZIP code. Individual-level data were obtained from the 2016 National Survey on HIV in the Black Community. We used multilevel modelling to estimate the association between willingness to use PrEP and clinic density among participants with individual-level (HIV risk, age, gender, education, income, insurance, doctor visit, census region, urban/rural residence) and ZIP code–level (%poverty, %unemployed, %uninsured, %black population, and density of health care facilities) variables.

**Results:**

All participants identified as black/African American. Of the 787 participants, 45% were men and 23% were found to be at high risk based on the self-reported behavioral characteristics. The mean age of the participants was 34 years (SD 9), 54% of participants resided in the South, and 26% were willing to use PrEP. More than one-third (38%) of the sample had to drive more than 1 hour to access a PrEP provider. Participants living in areas with higher PrEP clinic density were significantly more willing to use PrEP (one SD higher density of PrEP clinics per 10,000 population was associated with 16% higher willingness [adjusted prevalence ratio=1.16, 95% CI: 1.03-1.31]).

**Conclusions:**

Willingness to use PrEP was associated with spatial availability of clinics where providers prescribe PrEP in this nationally representative sample of black African Americans.

## Introduction

Black Americans comprise 44% of the nearly 40,000 individuals diagnosed with HIV in the United States in 2016 [1]. Pre-exposure prophylaxis (PrEP) in the form of tenofovir/emtricitabine is highly effective for the prevention of HIV [2,3]. Recent data suggest that although the need for PrEP is highest among black individuals, the rate of PrEP uptake is disproportionately low in this group compared to white Americans [4-6].

Individual-level barriers to PrEP use among black individuals include lack of awareness, low perceived HIV risk, and safety concerns [7-10]. PrEP also requires a prescription, and black individuals report a usual source of health care less often than white individuals [11]. Among black individuals, PrEP use may also be limited due to mistrust of the health care system, which is a result of contemporary and historical experiences of racism and discrimination [12,13].

Beyond these barriers, structural factors or aspects of one’s environment that are out of one’s immediate control, such as spatial access, may limit PrEP uptake [14-17]. Spatial access or proximity to services has been explored as a structural barrier to HIV services [18,19]. However, few studies have explored spatial access to PrEP. The purpose of this study was to determine the association between proximity to PrEP-prescribing clinics and willingness to use PrEP among black individuals in the United States.

## Methods

### Individual-Level Data

The National Survey on HIV in the Black Community was a cross-sectional survey administered to black individuals (aged 18 to 50 years) through Knowledge Networks, which is a probability-based, online, nonvolunteer Web panel [20]. Panel members were recruited from randomly selected addresses obtained from the US Postal Delivery Sequence File. All surveys were completed via email. Households without internet service were provided internet access.

To develop the survey, cognitive interviews were conducted with a convenience sample of black individuals, and 64 questions, one per screen, were included, some of which had automatic skip logic. A back button could be used to change responses. Once the survey was completed, the participant could not reenter the survey to submit additional surveys. A pretest was conducted with 26 cases and reviewed for accuracy. No personal information was collected. The median completion time was 13 minutes. No cookies were used, and duplication was not allowed in the panel.

This study was approved by Boston Children’s Hospital (Boston, MA). Informed consent was obtained prior to administration. Data were collected from February 12 to April 17, 2016. A US $5 online gift card was offered to all participants. All 1969 black participants in the panel were sampled. Of the 896 (45.50%) who provided consent, 868 (96.88%) were eligible and completed the survey. Only completed surveys were included in this analysis. The sample included individuals reporting high and low HIV risk. HIV-positive individuals were excluded. Post-stratification weighting was performed using sociodemographic benchmarks from the March 2016 supplement of the Current Population Survey [21] to ensure that estimates were representative of adults living in households in the United States in 2016 [22].

### Outcome

Willingness to use PrEP was ascertained by selecting yes, no, or maybe to item “If a pill that could prevent transmission of HIV from an HIV positive sex partner to an uninfected partner were available I would take it.” Responses were collapsed to obtain the risk ratio (yes vs no/maybe).

Based on prior research, the following covariates were selected: age (continuous); gender (male, female, or transgender male or female); HIV risk (more than one sex partner and no condom use in the last 3 months, anal sex with more than one partner and no condom use in the last 3 months, men having sex with men, history of a sexually transmitted infection in the last 3 months, drug use in the last 30 days, transgender individuals, and transactional sex work); education (less than high school, high school graduate/General Education Development [GED], college, or higher); income; insurance status (insured or uninsured); visits to a doctor (<12 months or >12 months); metropolitan statistical area (urban or rural); and region.

### Exposure

We obtained the locations of PrEP-prescribing clinics from AIDSVu.org [23] on September 29, 2017, for 760 unique ZIP codes and geocoded the addresses in ArcMap 10.4 [24], which produced 173 locations across 127 distinct ZIP Codes. The development and validation of the database of PrEP-prescribing locations has been described elsewhere [25,26]. We selected a random sample of clinics (10%) and confirmed the time of start of prescriptions to ensure the exposure date preceded collection of our outcome variable (willingness to use PrEP). We calculated the density of PrEP clinics per 10,000 residents according to the ZIP code by using the Census 2010 denominators [27] and the number of PrEP clinics per square mile using ArcMap 10.4 [24].

### ZIP Code Covariates

ZIP code–level covariates from the American Community Survey 2007-2011 (%living in poverty, %unemployed, %black population) and %uninsured from the American Community Survey 2008-2012 (unavailable for 2007-2011) were downloaded from the American Fact Finder Website [22]. We also adjusted for the density of clinics, community centers, and hospitals in 2016, which were retrieved from ERSI Business Analyst 2016 using North American Industry Classification System codes (621111 and 621112 for doctor offices, 621498 for community health care centers, and 622110 for hospitals) [23,28]. Kernel densities for each variable per ZIP code were created; we used principal component analysis to create one composite score, since the variables were highly correlated (Pearson *r*>0.84). Next, we created the variable driving distance (in miles) to the nearest PrEP clinic from the population-weighted centroid of all US ZIP codes within a 1-hour buffer, which incorporates street networks. Driving distance was measured from each geocentroid to the nearest PrEP-prescribing clinic. We limited the distance calculation to a 1-hour maximum to limit calculations that included crossing state lines. The variable was coded into four equal categories based on quartile distributions of drive times below 60 miles or <1 hour driving time, and the remainder of the sample was included within a fifth category, which served as the reference group. Drive time of more than 1 hour was chosen as the reference category, because a large proportion of the sample fell outside the 1-hour mark, which we assumed would be a barrier to accessing PrEP. Geospatial analyses were conducted using ArcMap 10.4 [24], and statistical analyses were performed using STATA 14.1 (Stata Corp., College Station, TX).

### Data Analysis

We merged the ZIP code data of individuals and subsequently conducted a multilevel, multivariable analysis to estimate the association between willingness to use PrEP and PrEP clinic density while adjusting for individual and ZIP code covariates in one block. Adjusted prevalence ratios (APR) were calculated along with the 95% CIs. Associations were considered significant at *P*<.05.

## Results

We included 787 participants and 700 distinct ZIP codes in the multilevel analysis. Among the participants, 45% were male and 23% were at high risk for HIV infection based on self-reported behavioral characteristics. The mean age of participants was 34 years (SD 9), 54% resided in the South, and 26% were willing to use PrEP. Among high-risk participants, 40.8% were willing to use PrEP. The mean number of PrEP clinics per ZIP code was 1.73 (SD 0.64), the density per 10,000 people was 0.07 (SD 0.22), and 38% of the sample had to drive more than 1 hour to access PrEP. Participants living in areas with higher PrEP clinic density were significantly more willing to use PrEP: 1 SD higher density of PrEP clinics per 10,000 people was associated with 16% higher willingness (APR=1.16, 95% CI=1.03-1.31). Participants with a high school diploma or GED were less likely to be willing to use PrEP than participants without such education levels (APR=0.60, 95% CI=0.37-0.99). Self-reported high HIV risk (APR=1.70, 95% CI=1.27-2.27) and residence in the West compared to Northeast (APR=2.04, 95% CI=1.06-3.93) were significantly associated with higher likelihoods of willingness to use PrEP (Table 1 and Table 2).

**Table 1:** Individual-level characteristics and multivariable associations with willingness to use pre-exposure prophylaxis (PrEP), 2016.

Individual-level characteristics included in the model (N=787)		Value	Adjusted prevalence ratio	95% CI
**Age (years), mean (SD)**		34 (9.03)	1.00	0.98-1.01
**Gender, n (%)^a^**				
	Male	308 (45.0)	1	Reference
	Female	479 (55.0)	1.16	0.87-1.54
**Education, n (%)**				
	Lower than high school	58 (11.0)	1	Reference
	High school diploma/GED^b^	169 (34.0)	0.60	0.37-0.99
	College or higher	560 (55.0)	0.74	0.46-1.19
**Income, n (%)**				
	<US $10,000	154 (16.0)	1	Reference
	US $10,000-$39,999	266 (29.0)	0.89	0.60-1.32
	US $40,000-$99,999	281 (41.0)	0.80	0.51-1.27
	≥US $100,000	86 (15.0)	0.67	0.34-1.34
**Insurance status, n (%)**				
	Currently insured	670 (83.0)	1.20	0.75-1.92
	Not insured	117 (17.0)	1	Reference
**Doctor visit, n (%)**				
	≤12 months	619 (77.0)	1	Reference
	>12 months or never	168 (23.0)	0.79	0.51-1.21
**HIV risk, n (%)**				
	Yes	194 (23.0)	1.70	1.27-2.27
	No	593 (77.0)	1	Reference
**Census region, n (%)**				
	Northeast	142 (19.0)	1	Reference
	Midwest	163 (18.0)	1.52	0.75-3.07
	South	389 (53.0)	1.45	0.77-2.74
	West	93 (11.0)	2.04	1.06-3.93
**Metropolitan statistical area, n (%)**				
	Urban	729 (91.0)	1	Reference
	Rural	58 (9.0)	0.72	0.38-1.37
**Willingness to use PrEP^c^, n (%)**				
	Yes	212 (26.0)	—^d^	—
	No/maybe	575 (74.0)	—	—

^a^Percentages are weighted.

^b^GED: General Education Development.

^c^PrEP: pre-exposure prophylaxis.

^d^Not available.

**Table 2:** ZIP code–level characteristics and multivariable associations with willingness to use pre-exposure prophylaxis (PrEP), 2016.

ZIP code–level variables included in model		Value	Adjusted prevalence ratio	95% CI
Density of PrEP^a^ clinics per 10,000 people, mean (SD)		0.00 (0.23)	1.16	1.03-1.31
**Driving distance to PrEP clinic from population centroid (miles)^b^, mean (SD)**		10.76 (11.77)	—^c^	—
	0-2.05	124 (15.76)	0.58	0.33-1.03
	2.06-7.19	131 (16.65)	0.70	0.43-1.11
	7.20-16.68	122 (15.50)	0.63	0.36-1.11
	16.69-57.27	114 (14.49)	0.70	0.45-1.10
	>1-hour drive time (reference)^d^	296 (37.61)	1	—
Density of doctors and outpatient clinics, mean (SD)		0.21 (0.24)	—	—
Density of CHCs^e^, mean (SD)		0.00 (0.00)	—	—
Density of hospitals, mean (SD)		0.01 (0.01)	—	—
Density of clinics/CHCs/hospital composites^f^, mean (SD)		—	1.04	0.80-1.33
Black/African American^g^ (%), mean (SD)		35.89 (28.52)	1.05	0.87-1.26
Unemployed^f^ (%), mean (SD)		11.42 (5.32)	1.05	0.84-1.31
Living in poverty^g^ (%), mean (SD)		20.02 (11.15)	0.98	0.75-1.27
Uninsured^f^ (%), mean (SD)		16.65 (6.79)	1.04	0.87-1.24

^a^PrEP: pre-exposure prophylaxis.

^b^This is a 5-level variable.

^c^Not available.

^d^Driving time is approximately 1 minute per mile. Therefore, >1-hour drive time is equivalent to 60 miles. This is the reference group.

^e^CHC: community health center.

^f^A composite variable created through principal components analysis using density of doctors/outpatient clinics, community health centers, and hospitals; higher scores indicate greater density.

^g^Mean weighted percentage.

## Discussion

Spatial proximity is a critical determinant of access to health services [29,30]. Our findings indicate that spatial access to PrEP-prescribing clinics is associated with greater willingness to use PrEP among black individuals. These findings provide additional evidence that access to PrEP must expand to increase uptake. In this study, spatial access may have led to greater willingness to use PrEP because of increased awareness through formal advertising, informal neighborhood networks, or direct knowledge of someone taking PrEP. Social capital and a general “culture of health” may also impact willingness and have been positively associated with use of HIV services [15,31].

Importantly, we found that nearly 40% of the sample would need to drive for >1 hour to access PrEP. Studies have shown that transportation barriers have a significant impact on health outcomes, particularly among disadvantaged individuals [32,33]. Regarding HIV care and treatment, travel time has been found to be a barrier to retention in care [34,35].

Based on our findings, an increase in the number of PrEP-prescribing providers in areas where access is currently limited would increase the use of PrEP. Recent studies have reported that many providers are unfamiliar with PrEP and have concerns about its safety and risk compensation [36-38]. Interventions that educate providers about PrEP are critical. Novel interventions such as the use of navigators or online prescriptions may also be necessary to increase uptake of PrEP.

This study has several limitations. Although the PrEP-prescribing database has undergone validation [25], sites may have been missed. In addition, the survey did not measure actual PrEP use. However, willingness to use PrEP provides a reasonable measure of potential uptake. Distance to PrEP sites could have been calculated from participants’ addresses; however, we only had access to participants’ ZIP codes. Although the sample size was modest, these data are nationally representative and weighted to reflect the population composition of black individuals in the United States.

In conclusion, this study demonstrates that black individuals with higher spatial access to PrEP-prescribing clinics were more willing to use this intervention. Scaling up of PrEP prescription at clinics in areas where black individuals reside is necessary to increase access to PrEP.

## Abbreviations

APR: adjusted prevalence ratios
CHC: community health center
GED: General Education Development
PrEP: pre-exposure prophylaxis
